# Another tool in the genome-wide association study arsenal: population-based detection of somatic gene conversion

**DOI:** 10.1186/1741-7015-9-13

**Published:** 2011-02-03

**Authors:** Matthew A Deardorff, Jesus Sainz, Struan FA Grant

**Affiliations:** 1 Division of Human Genetics, The Children's Hospital of Philadelphia Research Institute, Philadelphia, PA, USA; 2 Department of Pediatrics, The University of Pennsylvania School of Medicine, Philadelphia, PA, USA; 3 Institute of Biomedicine and Biotechnology of Cantabria (IBBTEC), Faculty of Medicine, University of Cantabria, Santander, Spain; 4 Spanish National Research Council (CSIC), Madrid, Spain; 5 Center for Applied Genomics, The Children's Hospital of Philadelphia Research Institute, Philadelphia, PA, USA

## Abstract

The hunt for the genetic contributors to complex disease has used a number of strategies, resulting in the identification of variants associated with many of the common diseases affecting society. However most of the genetic variants detected to date are single nucleotide polymorphisms (SNPs) and copy number variants (CNVs) and fall far short of explaining the full genetic component of any given disease. An as yet untapped genomic mechanism is somatic gene conversion and deletion, which could be complicit in disease risk but has been challenging to detect in genome-wide datasets. In a recent publication in *BMC Medicine *by Kenneth Ross, the author uses existing datasets to look at somatic gene conversion and deletion in human disease. Here, we describe how Ross's recent efforts to detect such occurrences could impact the field going forward.

See research article: http://www.biomedcentral.com/1741-7015/9/12/abstract

## Introduction

It is well established that genetic diversity combined with specific environmental exposures contribute to disease susceptibility. However, it has turned out to be challenging to isolate the genes underlying the genetic component conferring susceptibility to most complex disorders. The genetic underpinnings of such traits have remained largely unsolved until relatively recently, where the advent of array-based technologies and large population cohorts have enabled investigators to leverage genetic variation across the entire genome to pinpoint major contributing genetic factors. These discoveries have been primarily driven by genome-wide association studies (GWAS) using single nucleotide polymorphism (SNP) markers, which have revealed compelling evidence, including robust replication, for genetic variants associated with a broad range of phenotypes (see full catalogue at http://www.genome.gov/gwastudies).

These studies have been driven by arrays capable of estimating chromosomal quantitative data as well as SNP genotype status. As such, it has been possible to accurately genotype and rapidly quantify copy number variants (CNVs) [1-3], which have now been strongly implicated in common disorders such as autism [4-7], attention deficit hyperactivity disorder [8], schizophrenia [9-11] and childhood obesity [12].

Nonetheless, these approaches to date have generally only captured a small proportion of the predicted genetic component of various complex traits [13]. It is widely accepted that more extensive meta-analyses and high-throughput sequencing efforts with thousands of DNA samples from affected subjects could lead to further progress. However, these approaches will require large collaborative efforts and robust financial investment, respectively.

While advances are taking place on these fronts, the question remains of whether there are ways that the existing genome-wide SNP datasets could be mined further. After all, many datasets have been deposited in the public domain, most notably those found on dbGaP (http://www.ncbi.nlm.nih.gov/gap). The Wellcome Trust Case Control Consortium (WTCCC) has also made its datasets available to the wider scientific community and has been a key leader in whole genome genetic approaches [14,15].

In a study published this month in *BMC Medicine *[16], Kenneth Ross has made use of the WTCCC genome-wide SNP datasets for 7 common diseases, along with a shared pool of 3,000 controls to ask a focused but alternative question. Rather than looking for genetic polymorphisms residing in the germ line, he was interested in uncovering evidence of postzygotic somatic alterations, namely gene conversions and deletions, contributing to the pathogenesis of these diseases. Mitotic gene conversions have been shown to arise as a result of double-strand break repair that uses non-allelic homologous regions [17]. The effects of somatic gene conversion (see Glossary) have been shown to render genes non-functional, impact methylation status and aid the generation of deletions and other copy number variants; indeed, gene conversion has already been implicated in a number of disease settings [17-19].

The reason the approach described is so novel is that detecting these nearly identical recombinants has been technically difficult, due to both technological shortcomings faced by assessing close to identical sequences and difficulties associated with detecting such rare events in the face of a high background 'wild-type' signal.

Ross used the rationale that the genotyping data from most individuals in the WTCCC dataset were derived from blood, representing a population of cells, and that somatic gene conversion in an individual would result in a subtle shift of allele frequency data for an informative SNP. Since these relatively modest alterations can be difficult to detect at the individual level, he assessed whether there were statistical differences in the distribution of the frequency shifts between multiple control and disease populations. To help refine SNPs that were relevant to gene conversion, he used several additional strategies, including limiting analysis to those SNPs associated with regions of homology, and focusing on genotype frequencies that demonstrated unexpected deviation from Hardy-Weinberg equilibrium.

As a consequence of this study design, the author detected multiple instances of putative somatic gene conversion with duplicon identity. Although there is no experimental validation of the detected conversions, the author uses various metrics to assign relative strengths of certainty to the findings. He goes on to speculate on loci impacted by gene conversion and how they may be playing a role in disease.

Although the identified gene conversion is limited to blood, previous data has suggested that significant differences in sister chromatid exchange have been demonstrated in blood from patients with diseases in the WTCCC cohort [15]. Only one of the datasets was from lymphoblastoids and somewhat surprisingly these control samples did not show large differences from the blood genotyped controls.

This approach provides a new complementary methodology to detect gene conversion for regions where the CNV status has been previously characterized. This technique will, however, be somewhat more limited for variability still to be defined in specific individuals; indeed currently available genomic sequencing data suggests that such variability is extensive.

With these caveats in mind, and the fact that the analyses were limited to considering homologous regions, it is clear that this current study is primarily hypothesis forming, with various loci presented as potentially playing a role in disease risk. Nonetheless these hypotheses are testable, and the gene conversions identified by Ross can be tested in future datasets from DNA derived directly from target tissues or blood from other replication cohorts to further clarify their roles in these diseases. Once replicated, the field can move forward with greater certainty that perhaps at least one these gene conversion loci are contributing to disease risk and functional studies can be carried out to determine mode of action.

## Glossary

### Somatic gene conversion

This concept defines the process by which DNA sequence information is transferred in a non-reciprocal process from one genomic region to another region of the genome, altering its sequence. The transfer of genomic information is due to base mismatch repair during the recombination in somatic division

### Duplicons

These are duplicated genomic segments, also known as segmental duplications. These elements are large genomic segments of recent origin and nearly identical sequence present as low copy repeats. The length of duplicons can vary from 1 kb to hundreds of kb and have a high level of sequence identity (>90%)

## Competing interests

The authors declare that they have no competing interests.

## Authors' contributions

All authors contributed equally to this work.

## Pre-publication history

The pre-publication history for this paper can be accessed here:

http://www.biomedcentral.com/1741-7015/9/13/prepub
